# Zinc Transporters Serve as Prognostic Predictors and their Expression Correlates with Immune Cell Infiltration in Specific Cancer: A Pan-cancer Analysis

**DOI:** 10.7150/jca.87880

**Published:** 2024-01-01

**Authors:** Yanfen Liu, Lu Wei, Zhiyu Zhu, Shuyi Ren, Haiyang Jiang, Yufei Huang, Xiaoyu Sun, Xinbing Sui, Lijun Jin, Xueni Sun

**Affiliations:** 1 School of Pharmacy, Key Laboratory of Elemene Class Anti-Cancer Chinese Medicines; Engineering Laboratory of Development and Application of Traditional Chinese Medicines; Collaborative Innovation Center of Traditional Chinese Medicines of Zhejiang Province, Hangzhou Normal University, Hangzhou, Zhejiang 311121, China.; 2 Department of Gastrointestinal & Pancreatic Surgery, Key Laboratory of Gastroenterology of Zhejiang Province, Zhejiang Provincial People's Hospital, the Second Clinical College of Hangzhou Normal University, Hangzhou Normal University, Hangzhou, Zhejiang 311121, China.; 3 Department of Traditional Chinese Medicine, Hangzhou Shangcheng District People's Hospital, Hangzhou, China.

**Keywords:** trace element zinc, SLC30A, SLC39A, cancer prognosis, immune infiltration

## Abstract

The disruption of zinc (Zn) homeostasis has been implicated in cancer development and progression through various signaling pathways. Maintaining intracellular zinc balance is crucial in the context of cancer. Human cells rely on two families of transmembrane transporters, SLC30A/ZNT and SLC39A/ZIP, to coordinate zinc homeostasis. While some ZNTs and ZIPs have been linked to cancer progression, limited information is available regarding the expression patterns of zinc homeostasis-related genes and their potential roles in predicting prognosis and developing therapeutic strategies for specific cancers. In this study, a systematic analysis was conducted to examine the expression of all genes from the SLC30A and SLC39A families at both mRNA and protein levels across different cancers. As a result, three SLC39A genes (*SLC39A1*, *SLC39A4*, and *SLC39A8*) were found to be significantly dysregulated in specific cancers, including cervical squamous cell carcinoma and endocervical adenocarcinoma (CESC), liver hepatocellular carcinoma (LIHC), pancreatic adenocarcinoma (PAAD), and kidney renal papillary cell carcinoma (KIRP). Moreover, the dysregulation of these genes was tightly associated with the prognosis of patients with those cancers. Furthermore, we found that the gene *SLC39A8* exhibited the lowest mutation frequency in KIRP, whereas mutations in *SLC39A4* were found to significantly impact overall survival (OS), disease-free (DF), and progress-free survival (PFS) in cancer patients, particularly in those with PAAD. Additionally, immune infiltration analysis revealed that *SLC39A1*, *SLC39A4*, and *SLC39A8* may function as immune regulators in cancers. This provides new insights into understanding the complex relationship between zinc homeostasis and cancer progression.

## Introduction

Zn is a crucial trace element involved in various biochemical processes, and disruptions in intracellular Zn homeostasis have been associated with several pathological conditions. Previous studies have indicated the significance of Zn in antiviral activity and its role in respiratory viral infections 1, as well as its involvement in cancer development and progression 2. Dysregulated Zn levels are frequently observed in tumor tissues 3, 4. It was suggested that Zn may involve in cancer progression either by directly affecting cancer cells' proliferation and viability 5 or by regulating the tumor microenvironment 6. Two major groups of proteins, ZNT (SLC30A) and ZIP (SLC39A), are involved in regulating cellular Zn homeostasis. The SLC30A family comprises ten members (SLC30A1-10) responsible for exporting Zn^2+^ out of the cytoplasm, either to the extracellular environment or intracellular compartments 7. In contrast, the ZIP family comprises 14 members (SLC39A1-14) that facilitate the import of Zn^2+^ into the cytoplasm, leading to elevated cytosolic zinc concentrations 8.

Studies have consistently demonstrated that the expression of SLC30A/SLC39A family genes is closely linked to cancer progression. For example, differential expression of *SLC30A1* and *SLC30A6* has shown significant prognostic value in pancreatic cancer 9. *SLC39A4* shows significant upregulation in pancreatic cancer when compared to normal pancreatic tissues and has been proposed as a novel diagnostic marker for detecting the disease 10. The knockdown of SLC39A4 in pancreatic cancer cells leads to a significant inhibition of cell proliferation, migration, and invasion, indicating its potential as a therapeutic target 11. In lung cancer cells, silencing *SLC39A4* can inhibit cell migration and enhance sensitivity of lung cancer cells to cisplatin 8. The specific roles of *SLC39A14* and *SLC39A7* have also been well-documented in prostate cancer 12 and colorectal cancer cells 13, respectively, emphasizing the importance of zinc transporters in detecting specific cancers, predicting patient prognosis, and developing new anticancer therapies.

Given the critical roles of zinc transporters in cancer progression, this study aimed to systematically investigate the cancer-specific expression and prognostic value of these transporters through a pan-cancer analysis. The main objective was to explore the expression patterns of Zn homeostasis-related genes and their potential for predicting prognosis and developing therapeutic strategies for specific cancers. The analysis revealed that the expression of *SLC39A1*, *SLC39A4*, and *SLC39A8* is tightly associated with the prognosis of LIHC, CESC, PAAD, and KIRP, respectively. Additionally, the mutation analysis indicated that mutations in the *SLC39A4* gene have a significant and wide-ranging effect on DF, OS, and PFS in cancer patients, particularly in those with PAAD. Notably, *SLC39A4* was also identified as a potential immunomodulator in PAAD due to its strong correlation with immune cell infiltration in this cancer type.

## Materials and Methods

### Expression and prognostic value analysis of SLC30A/ SLC39A family genes in tumor tissues and cancer cell lines

The TissueNexus database, which integrates RNA-seq data from 52,087 samples of 49 human tissues/cell lines, was employed to analyze the expression of SLC30A and SLC39A family genes across 49 tissues/cell lines 14. This allowed for a comprehensive understanding of the expression patterns of SLC30A and SLC39A genes. To investigate the differential expression of these genes and their prognostic value in specific cancers, GEPIA online tool was employed. GEPIA provides a user-friendly interface for analyzing gene expression data and examining its correlation with patient survival. Additionally, the correlation between gene expression and patient survival was also verified through the UCSC Xena database, which contains comprehensive datasets for gene expression and clinical information. To further analyze the RNA-level expression of SLC30A and SLC39A family genes, the ALCAN database was utilized. To validate the survival curve, the standardized expression profile of pan-cancer in the UCSC Xena database and the corresponding clinical data were used. The standardized expression profile and clinical data of pancancer from the UCSC Xena data frame was downloaded. Tidyverse package was used to integrate the data, survminer and survival package were downloaded to make survival curves, and ggsurvplot package in R was used for data visualization.

### Biological functions investigation of *SLC39A1*, *SLC39A4*, and *SLC39A8* by Gene Set Enrichment Analysis

The LinkedOmics database was utilized to investigate the co-expression genes associated with *SLC39A1*, *SLC39A4*, and *SLC39A8*. The LinkedOmics database integrates clinical data from 32 cancers and includes information from 11,158 patients 15. To gain further insights into the biological functions of these co-expression genes, the GO and KEGG pathway enrichment analyses was performed through the DAVID database.

### Genomic alterations of *SLC39A1*, *SLC39A4*, and *SLC39A8* in cancers

The mutation status of *SLC39A1*, *SLC39A4*, and *SLC39A8* in various cancers and the impact of these mutations on clinical outcomes was analyzed by utilizing the cBioPortal database. The cBioPortal is a widely used web-based platform that allows for the exploration and analysis of cancer genomics data and provides valuable information on the genetic landscape of these zinc transporter genes in cancer and their potential impact on patient prognosis.

### Association analysis of *SLC39A1*, *SLC39A4*, and *SLC39A8* expression with immune cell infiltration in cancers

The TIMER database allows for the assessment of immune cell infiltration and the correlation with gene expression profiles in different cancer types, and was utilized to explore the correlations between the expression of *SLC39A1*, *SLC39A4*, and *SLC39A8* genes and immune cell infiltration in various cancers.

### qRT-PCR analysis

The differential expression of *SLC39A1* and* SLC39A4* in related normal cells and tumor cells was further evaluated by qRT-PCR. TRIzol was used to extract the total RNA. They were fluorescently stained with SYBR dye, and GAPDH was employed as the internal control. In addition, the expression of *SLC39A4* was examined at the RNA level in para-cancerous and tumor tissues of pancreatic cancer patients. Human tissue samples were provided by the Zhejiang Provincial People's Hospital under an approved protocol by the local medical ethics committee (2023-068). All patients were required to provide written informed consent. The following are the primers sequences: SLC39A1: Forward: 5'-GCTGTTGCAGAGCCACCTTA-3', Reverse: 5'-CATGCCCTCTAGCACAGACTG-3', SLC39A4: Forward: 5'-TGGTCTCTACGTGGCACTC-3', Reverse: 5'-GGGTCCCGTACTTTCAACATC-3', GAPDH: Forward: 5'-AACGGATTTGGTCGTATTGG-3', Reverse: 5'-TTGATTTTGGAGGGATCTCG-3'.

### Statistical analysis

Statistical differences between two groups were tested using the unpaired two-tailed t-test in Microsoft Excel. Statistical differences between more than two groups were tested using single factor analysis of variance (ANOVA) with Tukey's post-hoc HSD test. Differences were considered significant with a p-value < 0.05. Figures were prepared with GraphPad Prism 6 and are given as mean ± standard deviation (mean ± SD).

## Results

### Expression of SLC30A and SLC39A family genes in human tissues and cell lines

**Figure 1:**
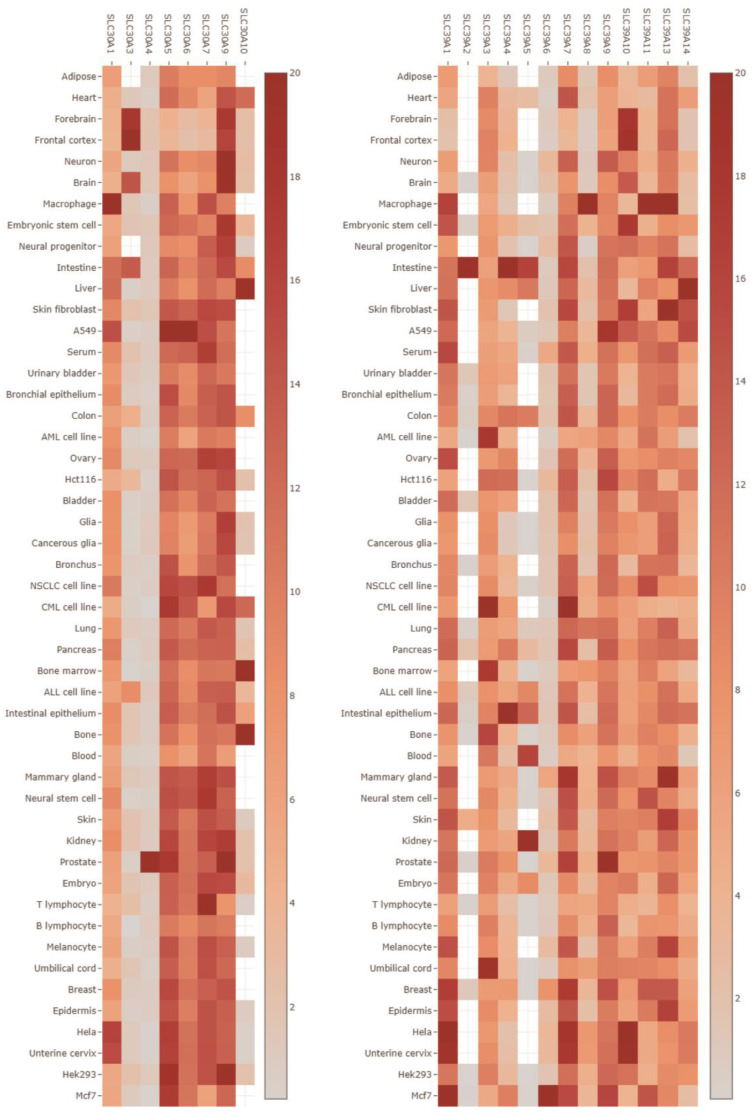
Expression of SLC30A/SLC39A families in 49 cells and tissues.

To investigate the tissue specificity of SLC30A and SLC39A family gene expression, we utilized the TissueNexus database. The analysis revealed that several genes within the SLC30A and SLC39A families, namely *SLC30A1*, *SLC30A5*, *SLC30A6*, *SLC30A7*, *SLC30A9*, *SLC39A1*, *SLC39A3*, *SLC39A7*, *SLC39A9*, *SLC39A10*, *SLC39A11*, *SLC39A13*, and *SLC39A14*, exhibit widespread expression in various tissues and cell lines such as the brain, intestine, liver, bladder, lung, pancreas, kidney, prostate, and breast (Figure 1). Conversely, *SLC30A3*, *SLC30A4*, *SLC30A10*, *SLC39A2*, and *SLC39A5* show limited or relatively low expression levels across most analyzed tissues and cell lines. Notably, our findings indicate that* SLC30A4* displays specific high expression in the prostate, while *SLC39A2* is exclusively expressed in the intestine (Figure 1), suggesting potential tissue-specific expression patterns for *SLC30A4* and *SLC39A2*. However, it should be mentioned that *SLC30A2*, *SLC30A8*, and* SLC39A12* were not included in this analysis due to unavailable information in the TissueNexus database.

Furthermore, we examined the protein-level expression of SLC30A and SLC39A family genes in 33 types of cancer as well as their corresponding adjacent tissues using GEPIA online tool. Our analysis demonstrated significant overexpression of *SLC39A1* (Supplementary Figure S1) in brain lower grade glioma (LGG), lymphoid neoplasm diffuse large B-cell lymphoma (DLBC), glioblastoma multiforme (GBM), testicular germ cell tumors (TGCT), thymoma (THYM), LIHC, and PAAD. Conversely, *SLC39A1* exhibited significant downregulation in KICH. The upregulation of *SLC39A4* (Supplementary Figure S1) was observed in bladder urothelial carcinoma (BLCA), breast invasive carcinoma (BRCA), CESC, colon adenocarcinoma (COAD), DLBC, esophageal carcinoma (ESCA), head and neck squamous cell carcinoma (HNSC), lung adenocarcinoma (LUAD), lung squamous cell carcinoma (LUSC), ovarian serous cystadenocarcinoma (OV), PAAD, rectum adenocarcinoma (READ), stomach adenocarcinoma (STAD), THYM, uterine corpus endometrial carcinoma (UCEC), and uterine carcinosarcoma (UCS). However, downregulation of SLC39A4 was observed in kidney renal clear cell carcinoma (KIRC), acute myeloid leukemia (LAML), and LGG. Additionally, *SLC39A8* (Supplementary Figure S1) exhibited significant overexpression in adrenocortical carcinoma (ACC), COAD, DLBC, ESCA, glioblastoma multiforme (GBM), KIRP, READ, STAD, TGCT, THYM, and UCEC, while its downregulation was observed in LUAD and LUSC. The differential expression profiles of other SLC30A and SLC39A family members, excluding *SLC39A1*, *SLC39A4*, and *SLC39A8*, across the 33 types of cancer were also analyzed, and the results are provided in Supplementary Figure S2-S3.

### Prognostic value assessment of *SLC30A* and *SLC39A* family genes in pan-cancer

**Figure 2:**
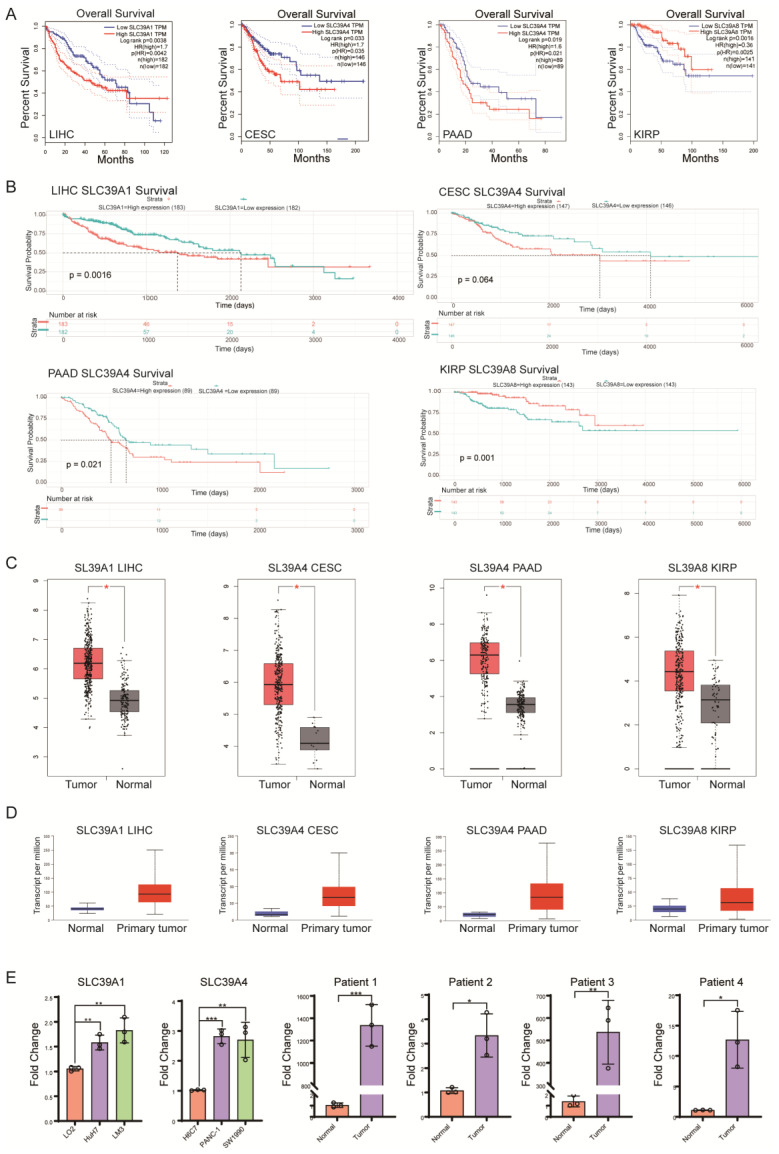
Expression of SLC39A1, SLC39A4 and SLC39A8. (A) Survival curves of SLC39A1 in LIHC, SLC39A4 in CESC, PAAD and SLC39A8 in KIRP. (B) R language was used to verify the OS of SLC39A1 in LIHC, SLC39A4 in CESC/PAAD and SLC39A8 in KIRP. (C) Gene expression of SLC39A1, SLC39A4 and SLC39A8 in normal and tumor tissues. (D) mRNA expression levels of SLC39A1, SLC39A4, and SLC39A8 in normal and tumor tissues. (E) mRNA expression levels of SLC39A1 and SLC39A4 in corresponding tissues and cells, 3 independent experiments (n=3), **p<0.01, ***p<0.001.

Subsequently, we evaluated the prognostic value of differentially expressed genes from the SLC30A and SLC39A families in the respective cancers. Initially, we analyzed the association between overall survival (OS) and the expression of SLC30A and SLC39A family genes across the 33 types of cancers. Notably, we observed a significant association between OS and the expression of *SLC39A1* in LIHC, *SLC39A4* in CESC and PAAD, and *SLC39A8* in KIRP. As depicted in Figure 2A, we performed a prognosis analysis of the SLC30A and SLC39A protein families in clinical patients using the GEPIA online tool. The results indicated that high expression of *SLC39A1* in LIHC (p=0.0042), high expression of *SLC39A4* in CESC (p=0.035) and PAAD (p=0.021), however low expression of *SLC39A8* in KIRP (p=0.0025), were associated with lower overall survival rates. These findings suggest the potential of *SLC39A1*, *SLC39A4*, and *SLC39A8* as prognostic markers for LIHC, CESC/PAAD, and KIRP, respectively.

To further validate the correlation between the expression levels of *SLC39A1*, *SLC39A4*, and *SLC39A8* and overall survival in LIHC, CESC/PAAD, and KIRP patients, we conducted Kaplan-Meier analysis using patient cohorts obtained from the TCGA database. Patient information is displayed in Supplementary Table S1. The results (Figure 2B) were consistent with the previous analyses, demonstrating that high levels of *SLC39A1* and *SLC39A4* predicted poor overall survival in LIHC (p = 0.0016) and CESC (p = 0.064)/PAAD (p = 0.021), respectively, while high levels of *SLC39A8* predicted favorable overall survival in KIRP (p = 0.001).

### The differential expression of *SLC39A1*, *SLC39A4*, and *SLC39A8* in normal and tumoral cell/tissues were validated using different data sources and laboratory experiments

Based on the above findings, we further validated the expression of *SLC39A1*, *SLC39A4*, and *SLC39A8* in LIHC, CESC/PAAD, and KIRP using different data resources. The results (Figure 2C) demonstrated that *SLC39A1* was highly expressed in LIHC patients, *SLC39A4* was highly expressed in CESC and PAAD patients, and *SLC39A8* was highly expressed in KIRP patients. Additionally, the mRNA expression of these genes was analyzed using the UALCAN database, which corroborated the protein-level expression analyses (Figure 2D).

Moreover, we conducted quantitative reverse transcription-polymerase chain reaction (qRT-PCR) analyses to corroborate the discernible variations in the expression of SLC39A1 within liver cancer cells (including primary human hepatocytes L-O2, HuH7, and LM3) and SLC39A4 in pancreatic cancer cells (comprising human pancreatic duct epithelial cell line H6c7, PANC-1, and SW1990). Additionally, the expression levels of SLC39A4 in paracancerous and tumorous tissues derived from pancreatic cancer patients were (Table 1) meticulously authenticated. Our findings, as illustrated in Figure 2E, unequivocally demonstrate that SLC39A1 exhibits significantly elevated expression in liver cancer cells compared to normal liver cells. Simultaneously, SLC39A4 manifests a notable upregulation in both pancreatic cancer cells and associated tissues. These results substantially reinforce the validity of our earlier observations.

### Genetic alteration analysis of *SLC39A1*,* SLC39A4*,* and SLC39A8* genes in specific cancer

In light of the aberrant expression and significant roles played by *SLC39A1*, *SLC39A4*, and *SLC39A8* genes in specific cancers, as well as the link between genetic alterations and cancer development, we further investigated the genetic alterations of these genes using the cBioPortal database. The analysis revealed that the mutation frequencies of *SLC39A1*, *SLC39A4*, and *SLC39A8* in pan-cancer patients were 4%, 6%, and 0.9%, respectively (Figure 2A). The primary types of genetic alterations observed for the genes *SLC39A1*, *SLC39A4*, and *SLC39A8* were amplification, missense mutation, and deep deletion. Notably, the genetic alteration rate of *SLC39A1* in LIHC reached 11%, while the rates of *SLC39A4* in CESC and PAAD were 3% and 10%, respectively, primarily characterized by amplification. Conversely, *SLC39A8* exhibited the lowest genetic alteration rate in KIRP, at only 1.1%, predominantly with missense mutations (Figure 2B).

We further analyzed the correlation between specific genetic alterations of these genes and patients' prognosis in pan-cancer. According to the Kaplan-Meier plotter analysis, among the 33 types of cancers examined, genetic mutation of *SLC39A1* was not found to have a significant association with prognosis of cancer patients (Supplementary Figure S4A). However, genetic mutation of *SLC39A4* had a significant impact on DFS and PFS in cancer patients (p-values: 2.718e-6 and 7.562e-4, respectively) (Supplementary Figure S4B). Additionally, genetic alteration of *SLC39A8* was closely correlated with OS in cancer patients (p-value: 0.0298) (Supplementary Figure S4C). Specifically, in LIHC, genetic mutation of *SLC39A1* showed no significant correlation with patients' prognosis (Figure 2C). However, mutations in *SLC39A4* had a significant effect on the OS, DFS, and PFS of PAAD patients, although no impact was observed in patients with CESC (Figure 2D-E). Due to the extremely small sample size, the correlation between genetic alteration of *SLC39A8* in KIRP and patients' prognosis could not be analyzed. These findings collectively indicate that genetic alteration of *SLC39A4* in PAAD may have a certain degree of impact on the prognosis of patients with pancreatic cancer.

### Biological function of *SLC39A1*,* SLC39A4*,* and SLC39A8* in related cancers

**Figure 3:**
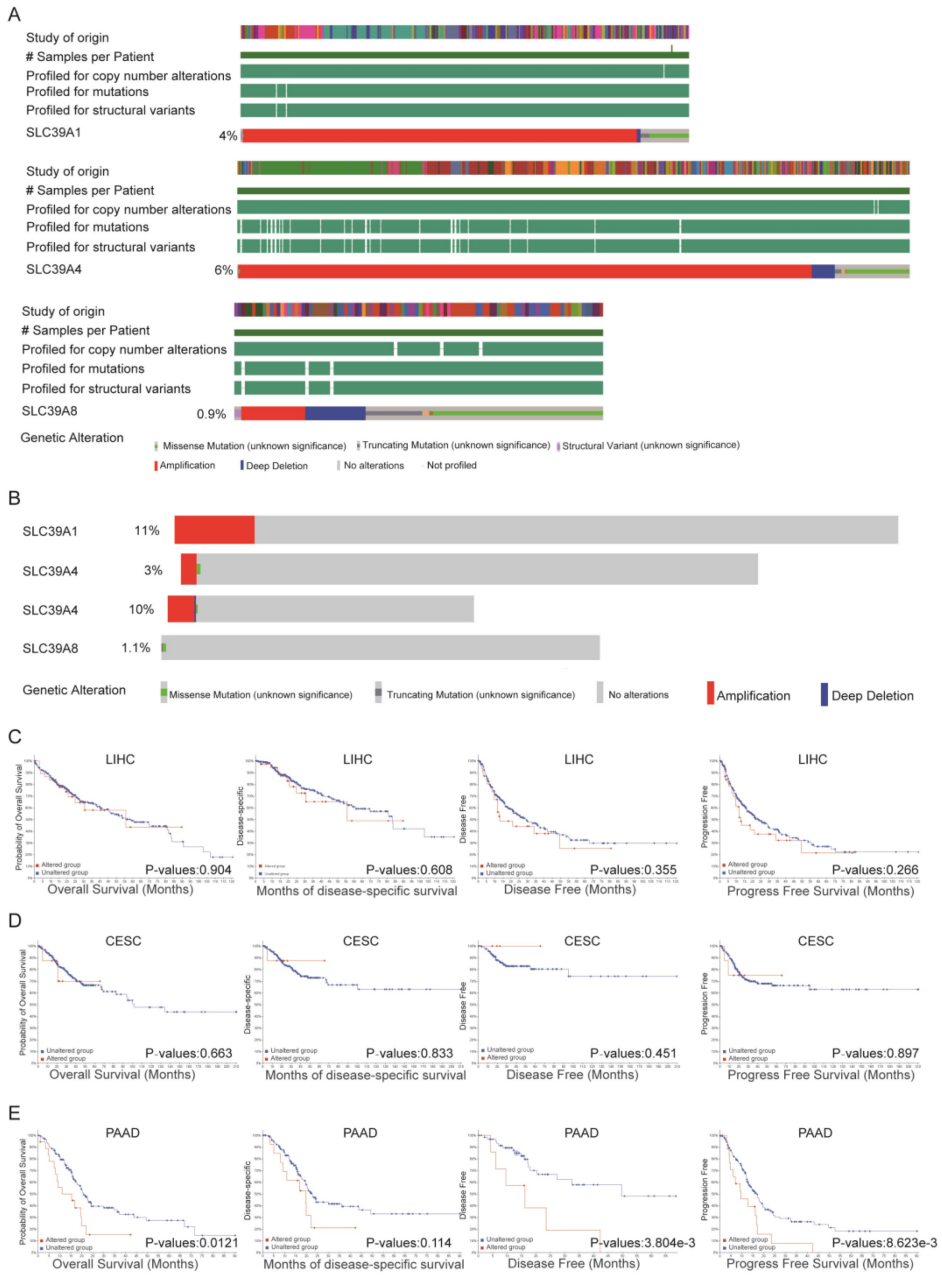
Mutations and prognosis significance of* SLC39A1, SLC39A4,* and *SLC39A8* in cancers*.* (A) Frequency of mutations in *SLC39A1, SLC39A4*, and *SLC39A8* in 33 types of cancers. (B) Mutation frequencies of *SLC39A1, SLC39A4,* and *SLC39A8* in LIHC, CESC/PAAD and KIRP, respectively. (C) Prognostic impact of *SLC39A1* mutation status on LIHC patients. (D) Prognostic impact of *SLC39A4* mutation status on CESC patients. (E) Prognostic impact of *SLC39A4* mutation status on PAAD patients.

To gain further insights into the biological functions of *SLC39A1*, *SLC39A4*, and *SLC39A8* in cancers, we utilized the LinkedOmics database to analyze their co-expression profiles in LIHC, CESC/PAAD, and KIRP, respectively. As a result, we obtained 19,921 genes related to *SLC39A1* in LIHC, 19,903 genes related to *SLC39A4* in CESC, 19,773 genes related to *SLC39A4* in PAAD, and 29,923 genes related to* SLC39A8* in KIRP (Figure 3A). The heat maps in Supplementary Figure S5 display the top 50 genes that positively correlate with *SLC39A1*, *SLC39A4*, and *SLC39A8*. We performed KEGG pathway enrichment analysis using the DAVID database with the top 20 positively correlated genes (P value < 0.001). In LIHC, the co-expression genes of *SLC39A1* were predominantly enriched in the Relaxin, Rap1, and FOXO signaling pathways (Figure 3B). In CESC, the genes associated with *SLC39A4* were primarily enriched in the PD-L1, Thyroid hormone, and T cell receptor signaling pathways (Figure 3C). Similarly, in PAAD, the *SLC39A4*-related genes were enriched in the same pathways (Figure 3D). Furthermore, the genes related to *SLC39A8* in KIRP were mainly involved in metabolic processes and the MAPK signaling pathway (Figure 3E). These findings indicate that *SLC39A1*, *SLC39A4*, and *SLC39A8* may play crucial roles in specific cancers by participating in various cellular processes and pathways.

However, ZIP proteins also play an important role in other cancers. ZIP1 is associated with chemotherapy resistance in lung cancer 16. It has antiproliferative effects on Prostate cancer 17 as well as effects on invasion and migration. In our study, ZIP1 serves as a potential prognostic marker in hepatocellular carcinoma. ZIP4 acts as an important regulator of the Snail-N-cadherin signaling axis in promoting non-small cell lung cancer progression 18. It has been shown that the expression level of ZIP4 is negatively correlated with the survival rate of hepatocellular carcinoma 19. In colon cancer, high expression of ZIP4 is associated with poorer prognosis in stage I-III patients 20. In the study herein, high expression of ZIP4 was highly correlated with low OS in pancreatic cancer. It has been claimed that ZIP8 is an important regulator of neuroblastoma cell proliferation and migration 21. However, in our study, ZIP8 was also found to be a potential prognostic marker for papillary cell carcinoma of the kidney.

### Correlation analyses of *SLC39A1, SLC39A4,* and *SLC39A8* expression with immune-related biomarker and immune cell infiltration in cancers

**Figure 4:**
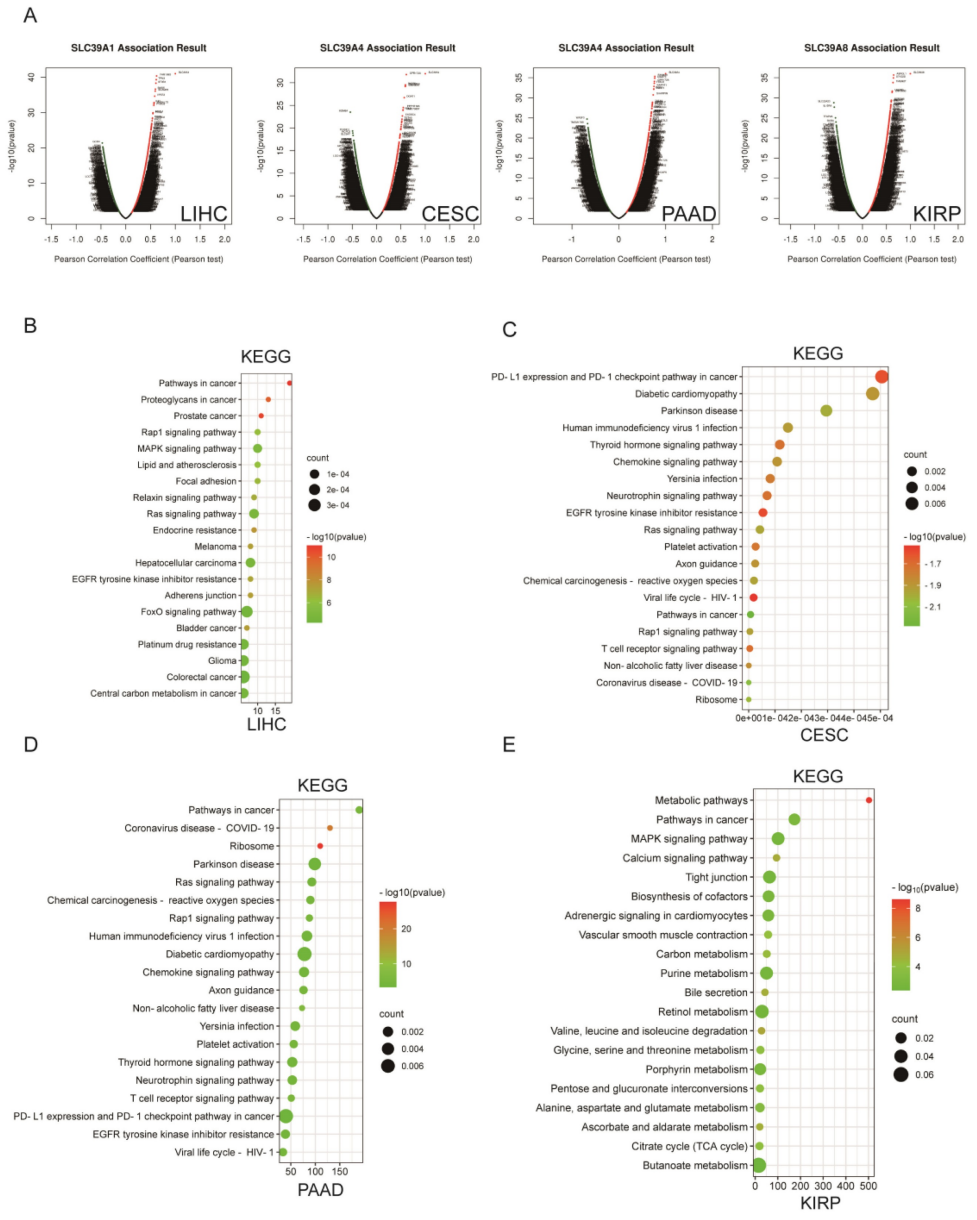
Biological function enrichment of *SLC39A1*, *SLC39A4*, and *SLC39A8* genes in corresponding cancers. (A) Volcano map of co-expressed profiling of *SLC39A1* in LIHC, *SLC39A4* in CESC/PAAD and *SLC39A8* in KIRP by the LinkedOmics database. (B) GO/KEGG analysis of genes co-expressed with *SLC39A1* in LIHC. (C) GO/KEGG analysis of genes co-expressed with *SLC39A4* in CESC. (D) GO/KEGG analysis of genes co-expressed with *SLC39A4* in PAAD. (E) GO/KEGG analysis of genes co-expressed with *SLC39A8* in KIRP.

**Figure 5:**
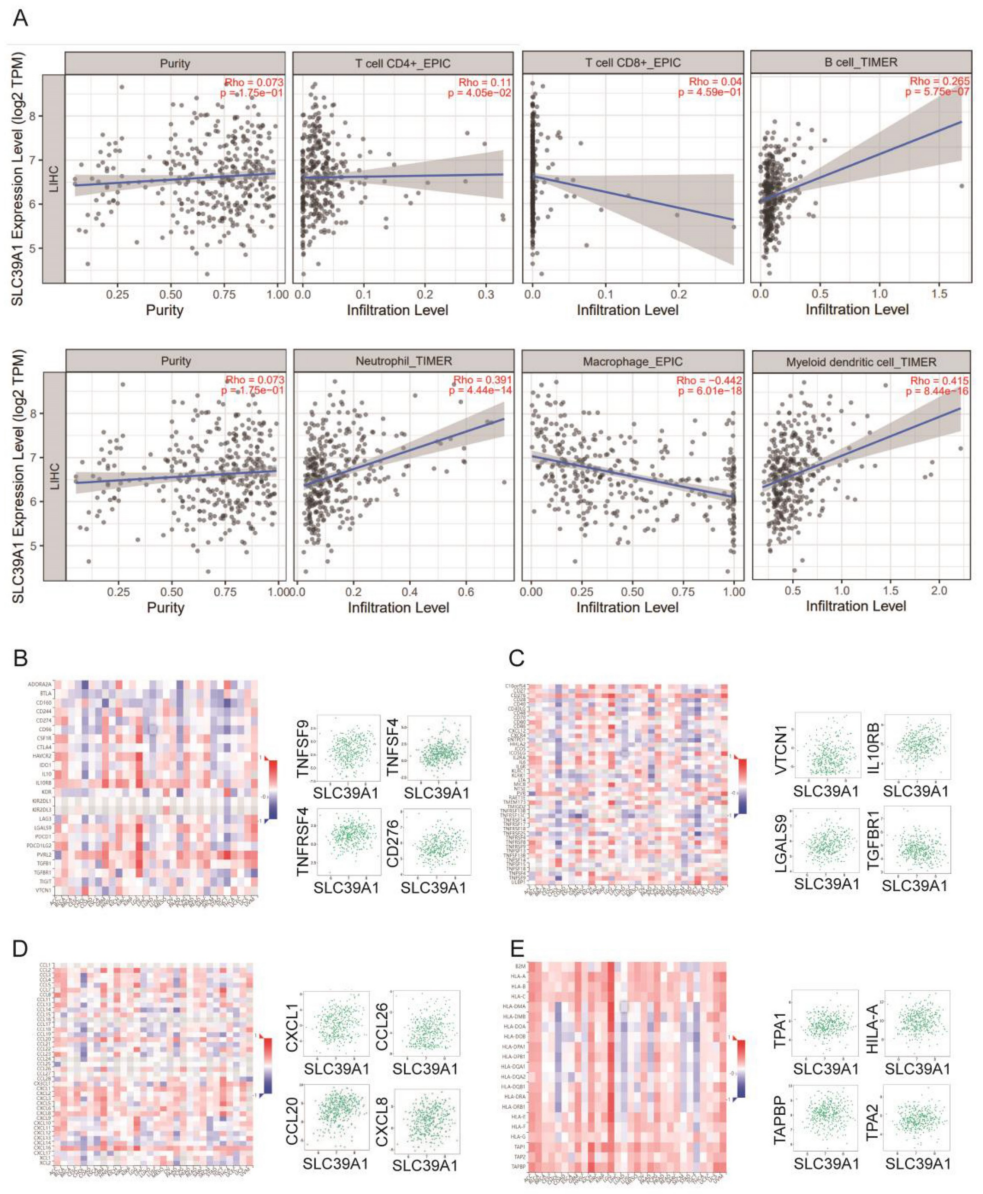
(A) Correlation between *SLC39A1* expression and the abundance of tumor infiltrating immune cells in LIHC available from the TIMER2.0 database. Correlation between *SLC39A1* expression and immunostimulators (B) and immunoinhibitors (C) in LIHC. Correlation between *SLC39A1* expression and chemokines (D) and chemokine receptors (E) in LIHC.

**Figure 6:**
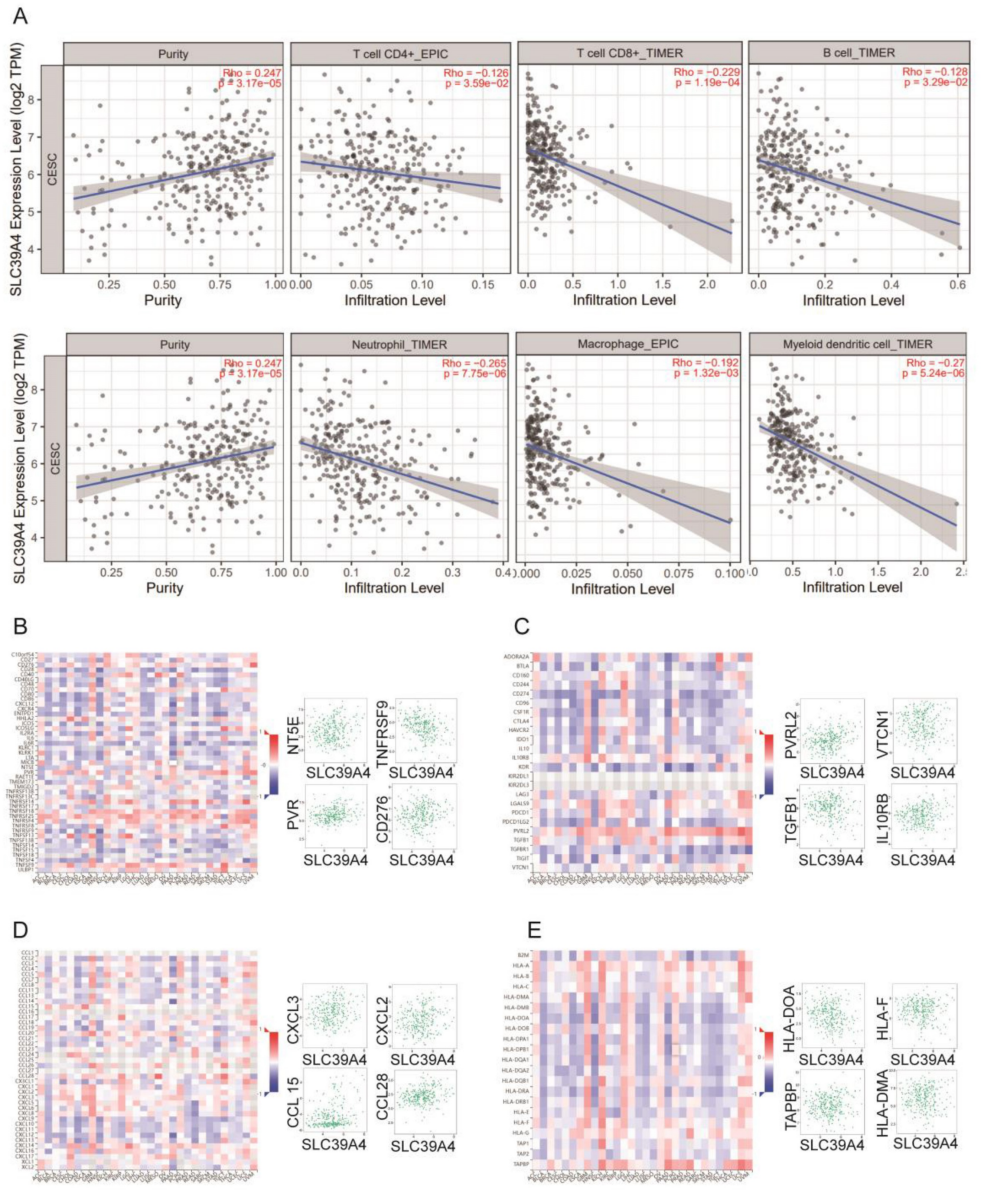
(A) Correlation between *SLC39A4* expression and the abundance of tumor infiltrating immune cells in CESC available from the TIMER2.0 database. Correlation between *SLC39A4* expression and immune-stimulators (B) and immune-inhibitors (C) in CESC. Correlation between *SLC39A4* expression and chemokines (D) and chemokine receptors (E) in CESC.

**Figure 7:**
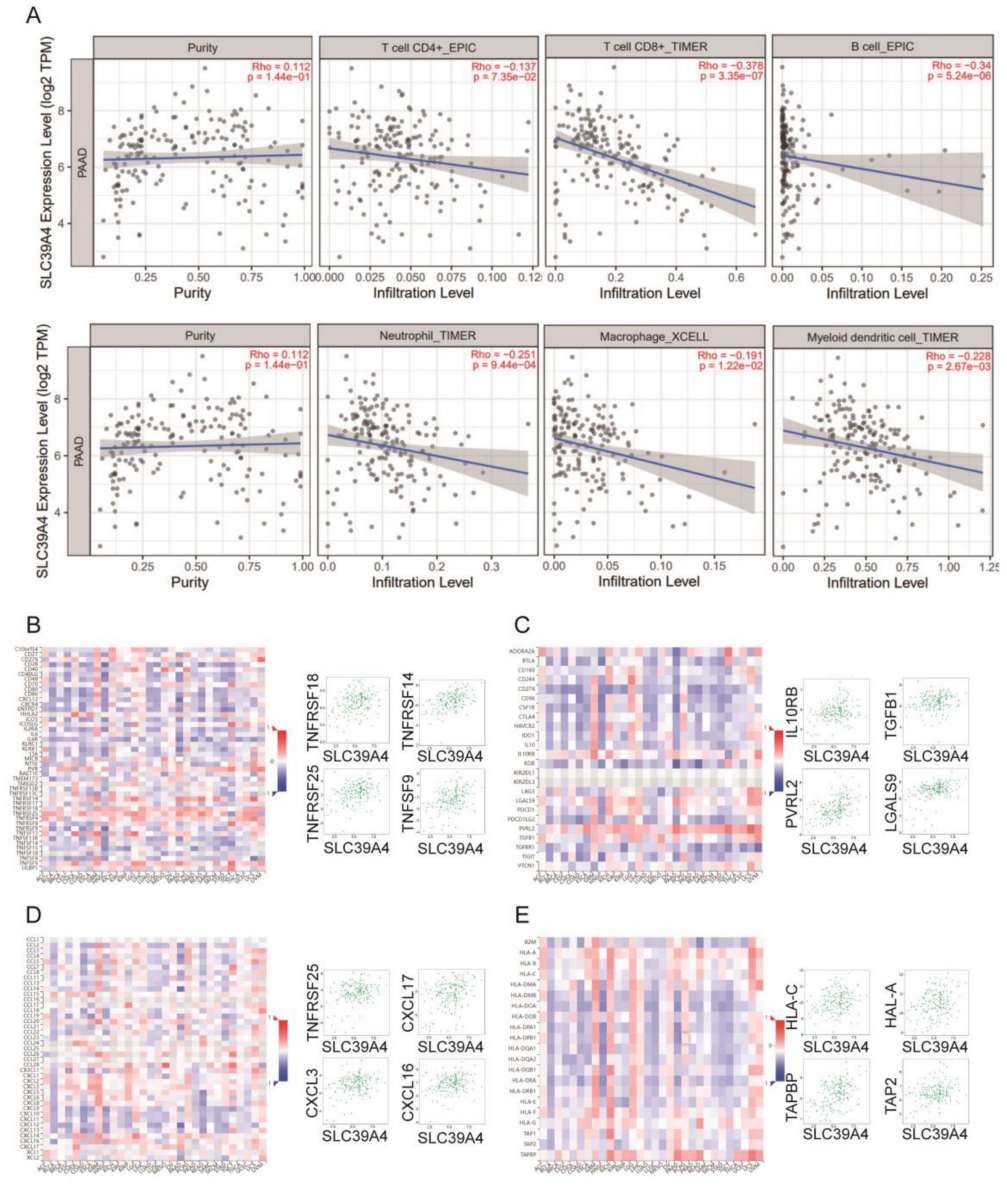
(A) Correlation between *SLC39A4* expression and the abundance of tumor infiltrating immune cells in LIHC available from the TIMER2.0 database. Correlation between *SLC39A4* expression and immune-stimulators (B) and immune-inhibitors (C) in PAAD. Correlation between *SLC39A4* expression and chemokines (D) and chemokine receptors (E) in PAAD.

Zn also plays a critical role in immunity as a catalytic and structural cofactor. Current studies have shown that zinc ions can regulate the function of T cells, monocytes, and macrophages, and can modulate immune responses through signaling pathways such as NF-κB 22, inspiring us to investigate the associations of Zn transporters expression and immune infiltration in related cancer. To investigate the associations between the expression of *SLC39A1*, *SLC39A4*, and* SLC39A8* and immune infiltration in related cancers, we utilized the TIMER database. We conducted an analysis to examine the relationship between the expression of these genes and the infiltration levels of immune cells, including B cells, CD4+/CD8+ T cells, myeloid dendritic cells, macrophages, and neutrophils. In LIHC (Figure 4A), *SLC39A1* expression showed a positive correlation with the infiltration levels of B cells (rho = 0.265, p = 5.75e-07), myeloid dendritic cells (rho = 0.415, p = 8.44e-16), and neutrophils (rho = 0.391, p = 4.44e-14). However, it was negatively correlated with the infiltration level of macrophages (rho = -0.442, p = 6.01e-18). In CESC and PAAD (Figure 5A and 6A), *SLC39A4* expression was negatively correlated with the infiltration of B cells, CD4^+^/CD8^+^ T cells, myeloid dendritic cells, macrophages, and neutrophils. Conversely, in KIRP (Figure 7A), the expression of *SLC39A8* exhibited a positive correlation with the infiltration levels of CD4^+^/CD8^+^ T cells, myeloid dendritic cells, and neutrophils, however, it showed a negative correlation with the infiltration levels of B cells and macrophages.

We further analyzed the correlation between the expression of *SLC39A1*, *SLC39A4*, and *SLC39A8* and immune-related factors, including immune-stimulators, immune-inhibitors, chemokines, and chemokine receptors. In LIHC (Figure 4B-E), *SLC39A1* expression exhibited positive correlations with several immune factors, such as TNFSF9, TNFSF4, TNFRSF4, CD276, VTCN1, IL10RB, LGALS9, TGFBR1, CXCL1, CXCL8, CCL20, CCL26, TPA1, TPA2, TAPBP, and HLA-A. In CESC (Figure 5B-E), *SLC39A4* expression showed positive correlations with immune factors like NT5E, TNFRSF9, PVR, CD276, PVRL2, VTCN1, TGFB1, IL10RB, CXCL2, CXCL3, CCL15, CCL28, TAPBP, HLA-DOA, HLA-F, and HLA-DMA. Similarly, in PAAD (Figure 6B-E), *SLC39A4* expression displayed positive correlations with immune factors such as TNFRSF18, TNFRSF14, TNFRSF25, TNFSF9, IL10RB, TGFB1, PVRL2, LGALS9, TNFRSF25, CXCL3, CXCL16, CXCL17, TAPBP, TAP2, HLA-C, and HLA-A. Notably, *SLC39A4* expression in PAAD showed a strongly negative association with most of the immune-inhibitors, indicating its potential role in regulating immune responses in PAAD. In KIRP (Figure 7B-E), *SLC39A8* expression positively correlated with immune factors such as CD40, CD70, HLA-A2, TNFSF13, VTCN1, CD160, HAVCR2, PVRL12, CCL21, CCL2, CCL11, CXCL12, HLA-DRB1, HLA-DOA, HLA-DPA1, and HLA-DRA. These findings suggest that *SLC39A1*, *SLC39A4*, and *SLC39A8* may play a role in modulating the immune environment in specific cancers. Their expression levels are associated with the infiltration of immune cells and correlate with immune-related factors, highlighting their potential as therapeutic targets in cancer treatment.

## Discussion and Conclusion

This study presents an investigation into the potential role of zinc transporters, specifically the SLC39A (ZIP) and SLC30A (ZNT) families, in cancer progression, along with their potential as prognostic markers and therapeutic targets. Zinc, as an essential trace element, plays a vital role in various biological activities, including structural stability, biocatalysis, and signal regulation 23, 24. Maintaining zinc homeostasis within the body is of utmost importance, as both zinc deficiency and excess can have detrimental effects on human health. Among the proteins associated with zinc homeostasis, zinc transporters, including members of the SLC39A and SLC30A families, play a vital role 25. These transporters facilitate the translocation of zinc ions in different directions, thereby maintaining the delicate balance of intracellular zinc ions. Aberrant expression of zinc transporters has been implicated in cancer progression, highlighting their potential significance in this context.

In this study, we conducted a comprehensive analysis of SLC30A and SLC39A family gene expression and mutation patterns across various cancer types. Moreover, the study explored the association between the expression levels of *SLC39A1*, *SLC39A4*, and *SLC39A8* and both prognosis and immune cell infiltration in respective tumors. Notably, *SLC39A1* demonstrated potential prognostic value in LIHC, while *SLC39A4* exhibited prognostic implications in CESC and PAAD. Clinically, liver transplantation is the only curative method and the 5-year survival rate after surgery is about 60% 26. It has been reported that the knockdown of *SLC39A1* can inhibit the proliferation of hepatocellular carcinoma cells and reduce the expression of cell cycle-related proteins 27, highly suggesting the vital role of *SLC39A1* in the progression of liver cancer. Besides, the previous study has shown that *SLC39A4* can be a prognostic marker for CESC 28, in agreement with our analysis in this study. The expression of *SLC39A4* was also linked to chemotherapeutic response in CESC. It was found that the knockdown of *SLC39A4* can significantly improve the sensitivity of CESC to cisplatin treatment 27. Moreover, we found that the genetic alteration rate of* SLC39A4* in PAAD is up to 10% and it is highly associated with a reduced OS in PAAD patients. This may partly explain the poor prognosis of PAAD patients. In contrast, *SLC39A8* displays the lowest mutation rate in cancers compared to *SLC39A1* and* SLC39A4*. The mutated *SLC39A8* is associated with a poor OS, disease-specific survival, and DFS of KIRP patients, indicating that the mutated *SLC39A8* may contribute to the increased mortality of this disease.

It was previously reported that in plants, SLC39A1 (ZIP1) acts as an immune signal peptide that activates cysteine proteases (PLCPs) to trigger the plant immune system and enhance plant resistance to pathogens 29. SLC39A8 was found to be specifically upregulated in CD4^+^ T cells that infiltrate inflamed joints. The deficiency of SLC39A8 in CD4^+^ T cells resulted in the abolishment of collagen-induced arthritis 30, suggesting a critical role for SLC39A8 in the development or progression of this autoimmune disease. This study revealed intricate relationships between the expression of zinc transporters and immune cell infiltration. For instance, *SLC39A1* and *SLC39A8* expression positively correlated with the infiltration of CD4^+^ T cells, neutrophils, and myeloid dendritic cells, along with the expression or release of immunosuppressants and activators. Conversely, *SLC39A4* expression in CESC and PAAD exhibited negative associations with the infiltration of CD4^+^/CD8^+^ T cells, B cells, myeloid dendritic cells, neutrophils, and macrophages. Notably, prior studies have reported the involvement of *SLC39A4* mutations, resulting in zinc deficiency and immune dysfunction 31. Therefore, a comprehensive investigation is warranted to comprehend the precise mechanisms by which zinc transporters modulate immune responses and the tumor microenvironment during cancer progression. The GO and KEGG enrichment analyses conducted in our study demonstrated the enrichment of zinc transporter-related genes in various biological processes, including cell metabolism, cell cycle regulation, and other essential processes. These findings are consistent with previous studies that have highlighted the importance of zinc transporters in these cellular processes 27, 32, 33.

However, additional pathological conditions afflicting patients may exert a substantial influence on the expression of zinc transporters. The human brain, being the organ with the highest zinc content, is particularly susceptible to perturbations in zinc concentration. An elevation in zinc levels within the brain can lead to neurotoxicity, while zinc deficiency is associated with various pathological manifestations, including malformations within the central nervous system. Investigation into postmortem brain tissues of individuals with Alzheimer's disease (AD) revealed heightened mRNA levels of the Zn^2+^ transporter protein ZIP1^34^. Such alterations in expression may not only reflect but also potentially contribute to modifications in cortical Zn^2+^ distribution in AD. Besides, studies have demonstrated that mutations in SLC39A4/ZIP4 result in acrodermatitis enteropathica. Certain mutations in mouse ZIP4, induced by acrodermatitis enteropathica, have been observed to impede plasma membrane transport. In specific mutants, ZIP4 tends to accumulate in the apical membrane, where diminished zinc uptake activity is evident due to a reduction in Vmax uptake 35. The indispensability of ZIP8 for the maintenance of normal liver function is underscored by findings indicating that moderate or acute reductions in ZIP8 activity induce pathological changes in the liver 36. Notably, the Zn^2+^ transporter protein ZIP8 exhibits specific upregulation in chondrocytes associated with osteoarthritis (OA), leading to heightened intracellular Zn^2+^ levels. This ZIP8-mediated Zn^2+^ efflux subsequently triggers an upregulation of chondrocyte matrix-degrading enzyme expression 37. Therefore, when exploring the relationship between zinc transporter expression and certain cancers, other diseases that may significantly affect zinc transporter expression should be better considered.

In conclusion, this study underscores the potential significance of zinc transporters, particularly *SLC39A1*, *SLC39A4*, and *SLC39A8*, as prognostic markers and therapeutic targets in various cancers. However, further validation and in-depth research are imperative to fully elucidate the underlying mechanisms and clinical implications of dysregulated zinc transporter expression in cancer. While this study provides valuable insights into the roles of zinc transporters in cancer, it is important to acknowledge its limitations, including the need for explicit exploration of the correlation between zinc ion concentration and zinc transporters in specific cancer types, as well as further validation of the observed associations using larger sample sizes and additional experimental and clinical investigations. Nonetheless, the findings underscore the potential of zinc transporters, particularly *SLC39A1*, *SLC39A4*, and *SLC39A8*, as promising prognostic markers and therapeutic targets in the field of oncology.

## Supplementary Material

Supplementary figures.

Supplementary table.

## Figures and Tables

**Figure 8:**
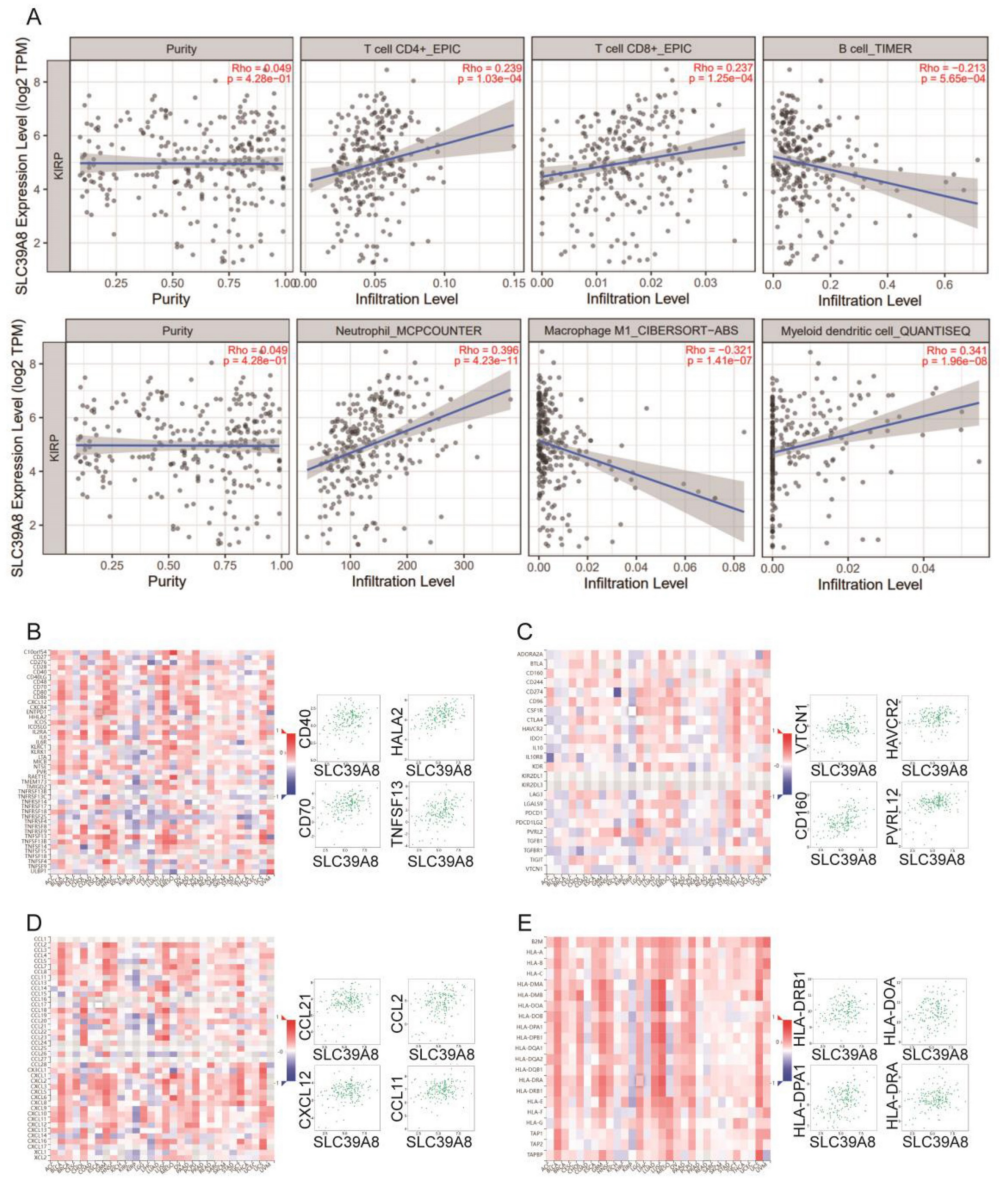
(A) Correlation between *SLC39A8* expression and the abundance of tumor infiltrating immune cells in LIHC available from the TIMER2.0 database. Correlation between *SLC39A8* expression and immune-stimulators (B) and immune-inhibitors (C) in KIRP. Correlation between *SLC39A8* expression and chemokines (D) and chemokine receptors (E) in KIRP.

**Table 1:** Patient information.

Patient ID	Sex	Age	Pathological type
Patient 1	Male	56	Moderate to poorly differentiated adenocarcinoma
Patient 2	Female	65	Moderately differentiated adenocarcinoma
Patient 3	Female	60	Poorly differentiated adenocarcinoma
Patient 4	Female	68	Moderately differentiated adenocarcinoma

**Table 2:** Roles of ZIP1, ZIP4, and ZIP8 reported in cancers.

Family member	Related Disease	Function	Ref.
ZIP1	Lung cancer	ZIP1^high^ stromal fibroblasts are associated with chemoresistance in lung cancer.	16
	Prostate cancer	Activation of specific intracellular signaling pathways for antiproliferative effects and effects on invasion and migration.	17
	Liver cancer	ZIP1 serves as a potential prognostic marker in hepatocellular carcinoma.	This study
ZIP4	Non-small cell lung cancer	ZIP4 acts as an important regulator of the Snail-N-cadherin signaling axis in promoting non-small cell lung cancer progression.	18
	Liver cancer	The expression level of ZIP4 is negatively correlated with survival in hepatocellular carcinoma.	19
	Colon cancer	A higher expression of ZIP4 is associated with a poorer prognosis for patients with stage I-III colon cancer.	20
	Pancreatic cancer	A higher expression of ZIP4 is highly associated with a lower OS in pancreatic cancer.	This study
ZIP8	Neuroblastoma	ZIP8 is an important regulator of neuroblastoma cell proliferation and migration.	21
	Kidney renal papillary cell carcinoma	ZIP8 serves as a potential prognostic marker in Kidney renal papillary cell carcinoma.	This study

## Abbreviations

ZNT: SLC30A
ZIP: SLC39A
CESC: Cervical squamous cell carcinoma and endocervical adenocarcinoma
LIHC: Liver hepatocellular carcinoma
PAAD: Pancreatic adenocarcinoma
KIRP: Kidney renal papillary cell carcinoma
OS: Overall Survival
DF: Disease-Free
PFS: Progress-free survival
GEPIA: Gene Expression Profiling Interactive Analysis
UCSC Xena: University of California, Santa Cruz
UALCAN: The University of Alabama at Birmingham
GO: Gene Ontology
KEGG: Kyoto Encyclopedia of Genes and Genomes
TCGA: The Cancer Genome Atlas Program
DAVID: the Database for Annotation, Visualization, and Integrated Discovery
LGG: Lower-grade glioma
DLBC: Diffuse large B-cell lymphoma
GBM: Glioblastoma multiforme
TGCT: Testicular germ cell tumors
BLCA: Bladder urothelial carcinoma
BRCA: Breast invasive carcinoma
COAD: Colon adenocarcinoma
ESCA: Esophageal carcinoma
LUAD: Lung adenocarcinoma
LUSC: Lung squamous cell carcinoma
OV: Ovarian serous cystadenocarcinoma
READ: Rectum adenocarcinoma
STAD: Stomach adenocarcinoma
UCEC: Uterine corpus endometrial carcinoma
UCS: Uterine carcinosarcoma
KIRC: Kidney renal clear cell carcinoma
LAML: Acute myeloid leukemia
ACC: Adrenocortical carcinoma
