# Supplementary material for: Zinc Transporters Serve as Prognostic Predictors and their Expression Correlates with Immune Cell Infiltration in Specific Cancer: A Pan-cancer Analysis
Source: J Cancer. 2024 Jan 1;15(4):939–54. doi: 10.7150/jca.87880 (PMC10788725; doi:10.7150/jca.87880)
Supplement: Supplementary file 1 — Supplementary figures. [file jcav15p0939s1.pdf]

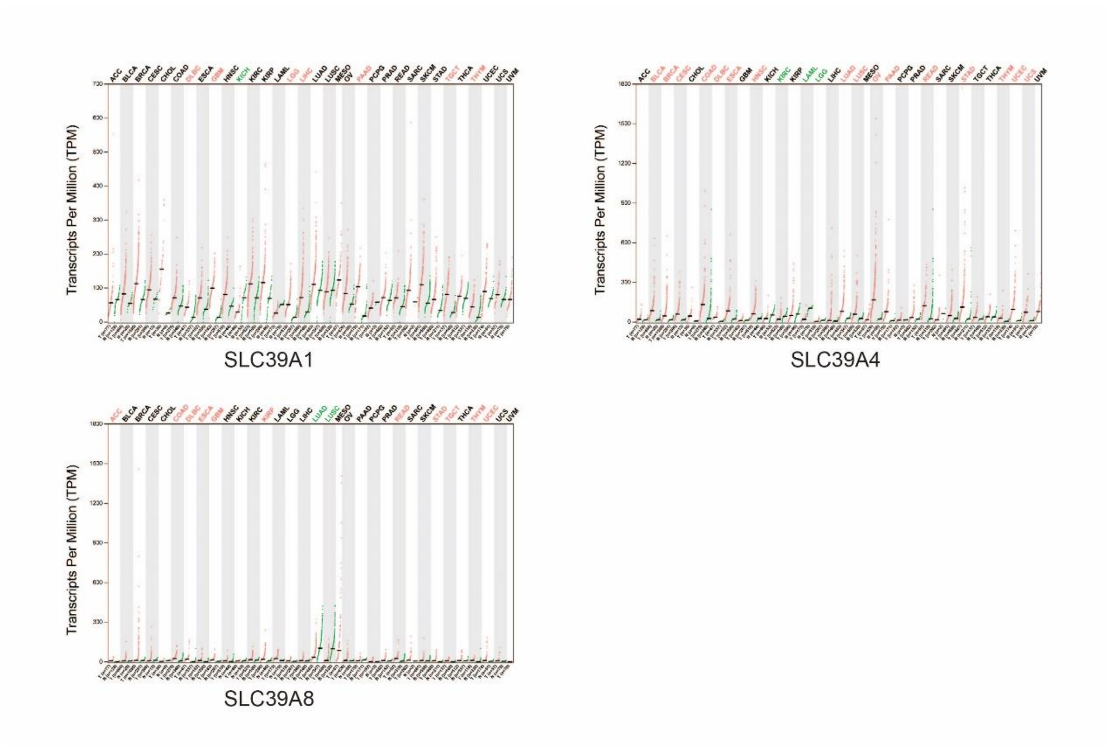

Figure S1. Expression of *SLC39A1*, *SLC39A4*, and *SLC39A8* in 33 types of cancers.

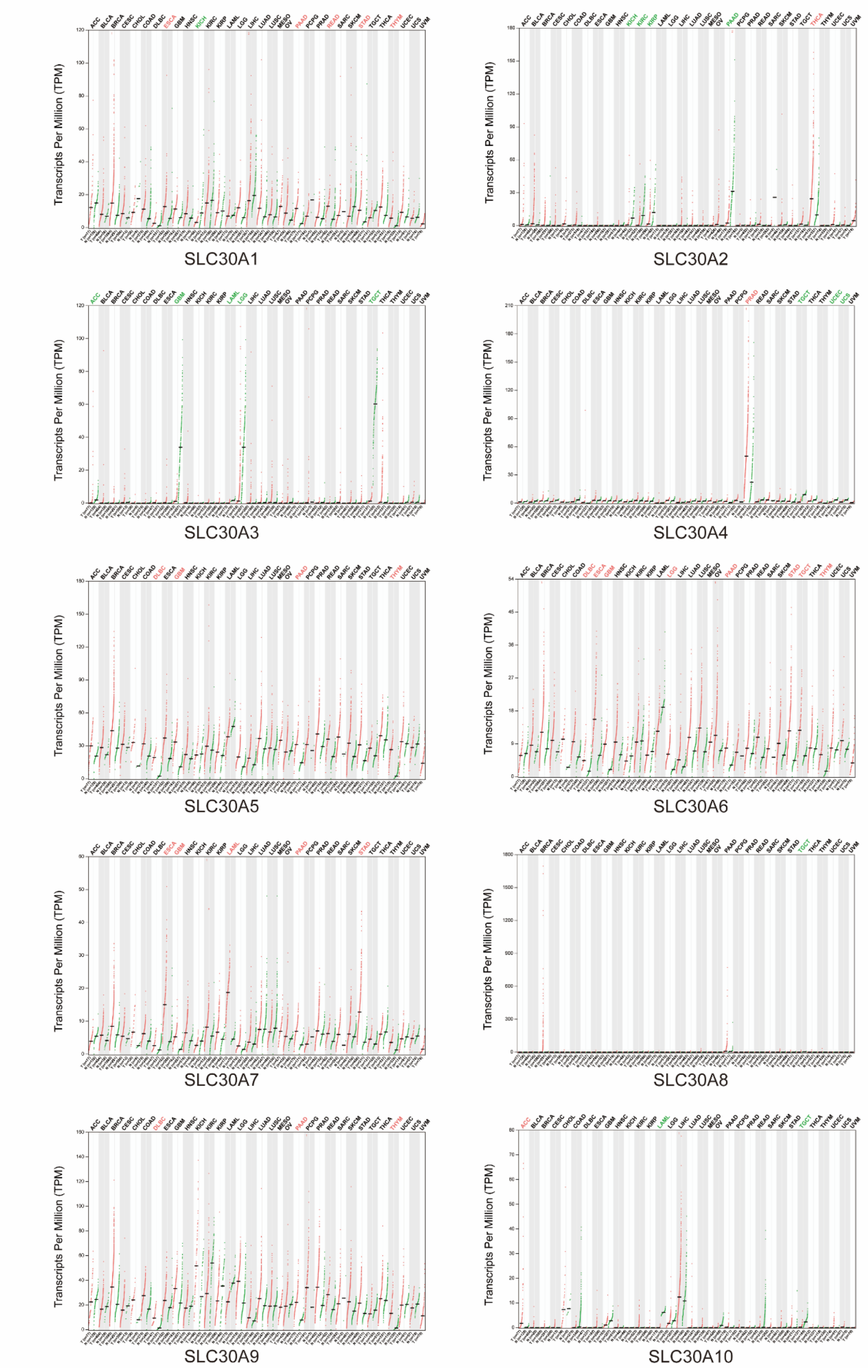

Figure S2. Expression of SLC30A families in 33 types of cancers.

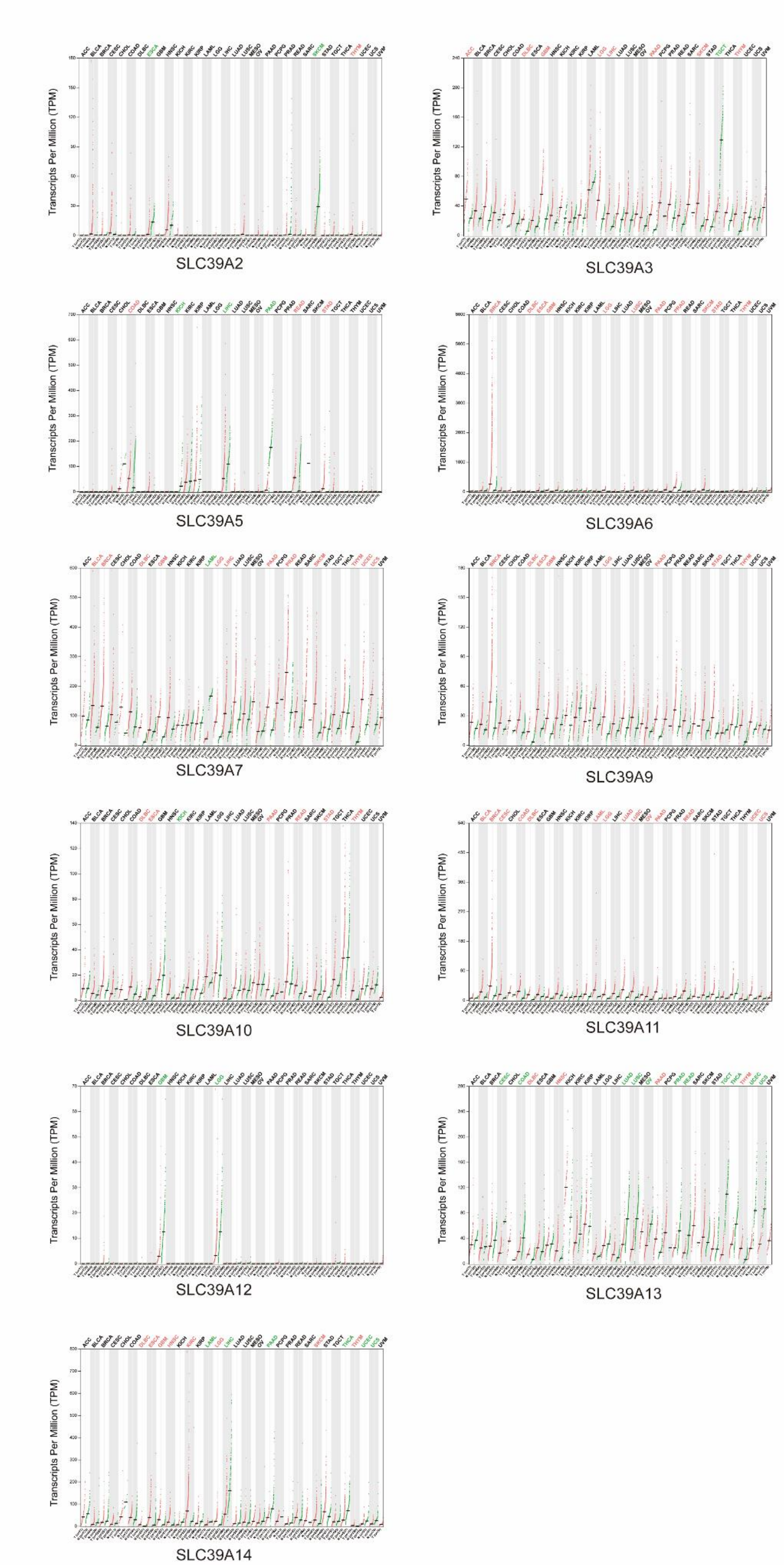

Figure S3. Expression of SLC39A families in 33 types of cancers.

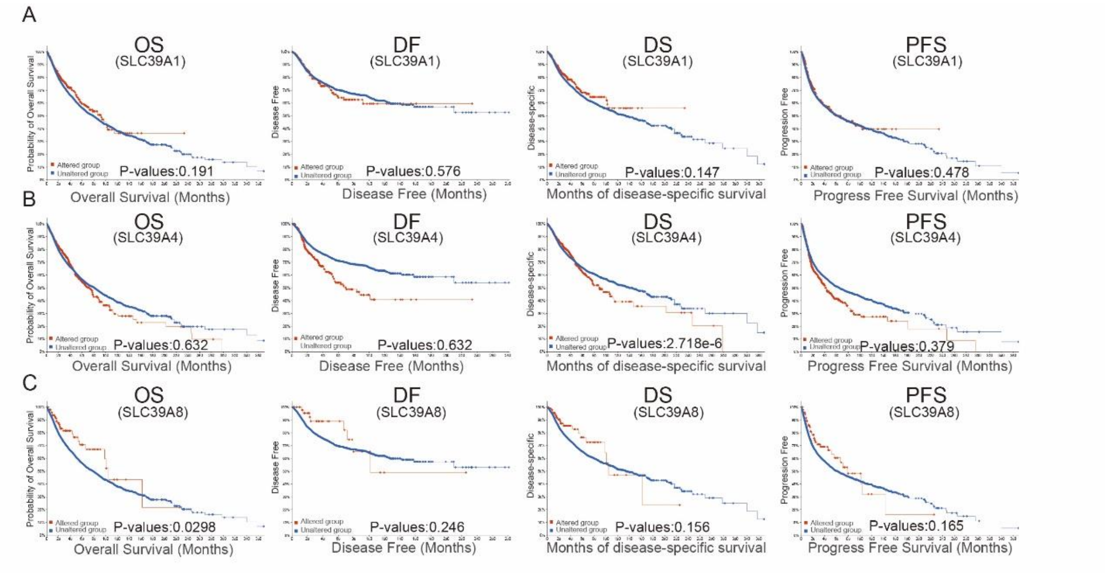

Figure S4. OS, DF, DS, PFS of cancer patients with or without *SLC39A1* mutation (A). OS, DF, DS, PFS of cancer patients with or without *SLC39A4* mutation (B). OS, DF, DS, PFS of cancer patients with or without *SLC39A8* mutation (C).

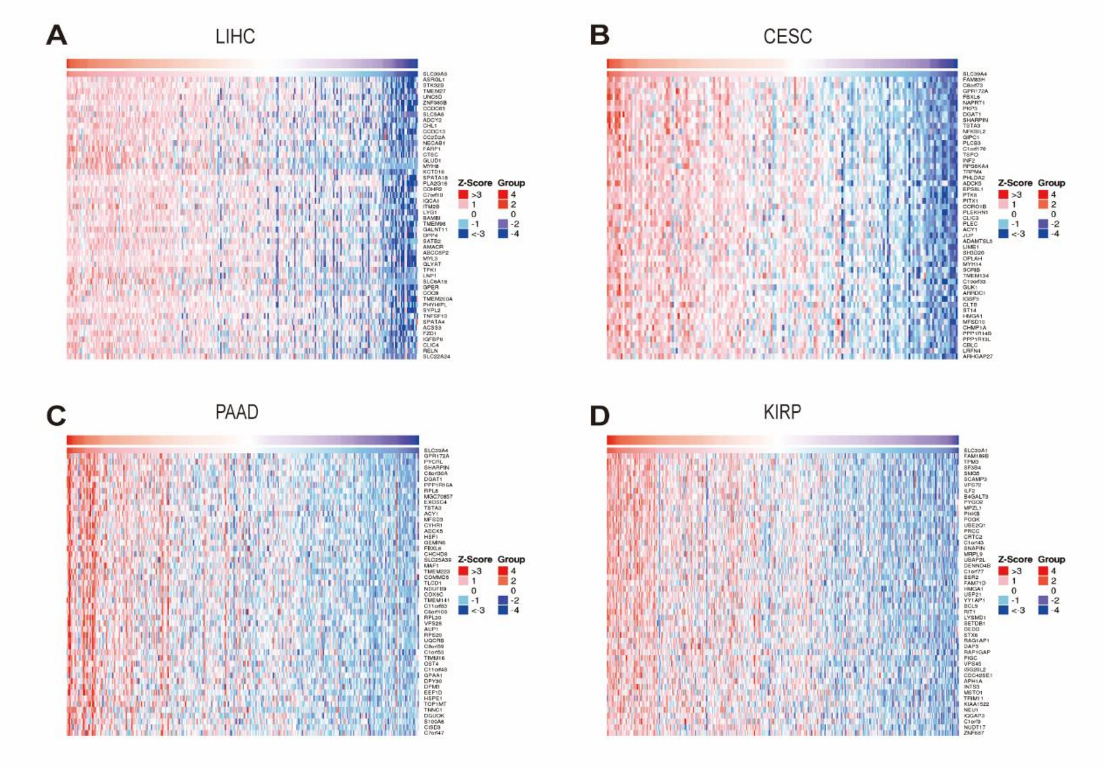

Figure S5. Top 50 genes positively correlated with *SLC39A1* expression in LIHC (A). Top 50 genes positively correlated with *SLC39A4* expression in CESC (B). Top 50 genes positively correlated with *SLC39A4* expression in PAAD (C). Top 50 genes positively correlated with *SLC39A8* expression in KIRP (D).
